# The clinical impact of miRNA34a and P53 gene expression in colon cancer

**DOI:** 10.1016/j.bbrep.2018.10.002

**Published:** 2018-10-24

**Authors:** Eman A.E. Badr, Mohamed Farag Ali Assar, Suzy F. Gohar, Mohamed Hamdy Badr, Rawda Magdy Hathout, Salah Mohamed El-kousy

**Affiliations:** a Department of Medical Biochemistry and Molecular Biology, Faculty of Medicine - Menoufia University, Egypt; b Department of Chemistry, Biochemistry Division, Faculty of science - Menoufia University, Egypt; c Department of Clinical Oncology, Faculty of Medicine - Menoufia University, Egypt; d Department of Internal Medicine, Faculty of Medicine - Menoufia University, Egypt; e Chemist at Faculty of Science – Menoufia University, Egypt; f Department of Organic chemistry, Faculty of science - Menoufia University, Egypt

**Keywords:** Cancer – colon/rectal, Cell – biology, Clinical research

## Abstract

**Objective:**

To study the potential role of miRNA34a gene expression and its relationship with P53 gene expression, fate, stage, metastasis and overall survival of colorectal cancer.

**Patients and methods:**

This study was carried out 30 patients with colon adenocarcinoma, 30 patients with benign colon polyp and 30 apparently healthy persons served as controls. All participants were subjected to full history taking, general clinical examination. Complete blood count, liver and kidney function, determination of serum tumor markers were done. Estimation of microRNA 34a and P53 Gene expression by real-time PCR were done.

**Results:**

There was a significant negative relationship between serum tumor markers and micro RNA 34a gene expression in cancer patients. Also, there was a statistically significant positive relationship between miRNA34a gene expression and P53 gene expression in both patients groups. The diagnostic accuracy of miRNA34a gene expression was both sensitive and specific for colon cancer. MiRNA34a and P53 gene expression had statistically significant relation with tumor stage and presence of metastases.

**Conclusion:**

It can be concluded that the level of miRNA34a can be used to differentiate between colon cancers and begin adenomas. MiRNA34a can be used as a prognostic marker in colon cancer.

## Highlights

• MiRNA34a has an important role in colon cancer.
• Presence of correlation between P53 and miRNA34a gene expression.
• MiRNA34a can be used as a prognostic marker in colon cancer.
• MiRNA34a can be used to differentiate between colon cancer and benign adenoma.

## 1 Introduction

Colorectal cancer classified worldwide as the third common cause of cancer and the fourth cause of cancer deaths [1].

Colonoscopy is an invasive and uncomfortable method but it still considers as the most reliable screening method for diagnosis of colorectal cancer. While other markers as the fecal occult blood test, serum CA19-9 and CEA have either low sensitivity, specificity or both [2].

So, there may be a necessary need for another noninvasive biomarker for colorectal cancer diagnosis like miRNAs which approved to have a role in its oncogenesis [3].

MiRNAs are 21–25 nucleotides non-coding RNAs which may be related to cell proliferation, differentiation, and apoptosis [4].

Different miRNA may pose tumor suppressor or oncogenic properties with altered gene expression in cancerous tissue in comparison to the normal one [5].

The miRNA34 family consists of miRNA34a, miRNA34b, and miRNA34c, which has a tumor suppressive property due to involvement in cell cycle and apoptosis. [6].

MiRNA 34a gene transcription influence by P53 level. In turn, the level of miRNA34a gene expression may affect the P53 level by stimulation of P53 promoter. [7], [8].

Alterations in different molecular signaling pathways are involved in the initiation and progression of colon cancer. An example of those pathways is Notch-1, Notch-2 pathway which suppressed by up-regulation of miRNA34a gene expression [9].

MiRNA-34a affect the Notch signaling by binding to 3` UTR of messenger RNA sequences of Notch receptors causing a reduction in Notch protein levels. This pathway plays an important role in self- renewal, and differentiation of colon stem cells. So, miRNA34a can influence oncogenesis and cell fate determination [10], [11].

The micro RNA (miRs) may be used as a potential therapeutic target; this can be achieved by administration of synthetic miRs or administration of miR-expressing vectors. MiRNA-34a-based replacement therapy is a promising approach in cancer treatment [12].

This study aims to investigate the potential role of miRNA34a gene expression as biomarkers of colorectal cancer, whereas it is diagnostic or prognostic, their relationship with P53 gene expression, fate, stage, metastasis and overall survival of colorectal cancer.

## 2 Subjects and methods

### 2.1 Subjects

This study was carried out by cooperation between Medical Biochemistry, Internal Medicine and Clinical Oncology Departments, Faculty of Medicine, Menoufia University.

It included ninety individuals: 60 Patients and 30 age and gender-matched healthy subjects selected from Internal Medicine and Clinical Oncology Departments and outpatient clinics, Menoufia University Hospital in the period from June 2016 to August 2017. The studied subjects were categorized into the following three groups:

Group I: included 30 patients with histo-pathologically proved colonic adenocarcinoma.
Group II: included 30 patients with histo-pathologically proved benign colonic polyps (colorectal adenoma).
Group III: Included 30 ages and gender-matched apparently healthy individuals as a control group.

Patients with preoperative chemo or radiotherapy, history of inflammatory bowel disease, or hereditary nonpolyposis colorectal cancer (HNPCC) were excluded from the study.

An informed written consent was obtained from all subjects participated in this study. The protocol was approved by the Ethical Committee of Medical Research, Faculty of Medicine, Menoufia University.

All participants were subjected to: Full history taking, clinical examination, body mass index (BMI) was estimated for all participants.

Laboratory investigations including: Complete blood count, liver and kidney function, determination of serum carbohydrate antigen 19-9 (CA19-9) and carcinoembryonic antigen (CEA) levels were done.

The baseline blood samples were obtained from all participants for Estimation of microRNA34a and P53 Gene expression by real-time PCR.

Colorectal cancer patients underwent baseline computed tomography of chest, abdomen and pelvis and bone scan for initial staging evaluation and were staged based on the TNM classification and the pathological grading was based on the world health organization (WHO) criteria and performance status was estimated based on ECOG classification [13].

All patients with stage III disease underwent surgical resection followed by adjuvant 6 cycles of FOLFOX4 regimen and were initially evaluated by CTs and tumor markers after 3 cycles.

Only 2 patients with stage II disease were presented with intestinal obstruction and were received adjuvant treatment and evaluated in the same way as patients with stage III disease. While, the rest of patients with stage II (5 patients) underwent surgery and kept under follow up by CTs and tumor marker every 3 months and annual colonoscopy.

Patients with metastatic (stage IV disease) the main treatment was systemic chemotherapy using FOLFOX4 regimen and also were initially evaluated after 3 cycles of chemotherapy by CTs and tumor markers.

All patients were followed up for at least 12 months (12–14 months).

Response to treatment was assessed according to response evaluation for carcinoma in solid tumors (RECIST) criteria. [14] .

### 2.2 Methods

#### 2.2.1 Blood sample

Five ml of venous blood were withdrawn by venipuncture; 3 ml was transferred into a plain tube, left to clot, centrifuged for 10 min at 4000 R.P.M. The serum obtained was stored at −80 ºC until analysis of serum CA19-9 and CEA. The remaining 2 ml of blood were collected into EDTA containing tube for RNA extraction & estimation of Gene expression of miRNA34a and P53 by using real-time PCR.

Serum CA19-9 and CEA were determined by enzyme-linked immunosorbent assay method, using Human CA19-9 ELISA kit, Chemux BioScience, Inc, USA.

Assay of microRNA 34a and P 53 gene expression

Total and microRNA was extracted from whole blood by Direct-zol™ RNA MiniPrep kit, Zymo Research.

Two-step RT–PCR was done as follows:

For miRNA34a gene expression

For the preparation of RT Master Mix: 1.5 μl RT buffer, 0.15 ul dNTP Mix, 3 μl RT primer, 1 μl MultiScribe™ Reverse Transcriptase, 0.19 RNase Inhibitor, 4.16 Nuclease-Free Water were pipetted into each well. 5 μL of RNA sample was pipetted into each well and pipetted up and down two times to mix. Then the samples were incubated at 16 °C for 30 min and at 42 °C for 30 min. Heating to 85 °C for 5 min for inactivation of the reverse transcriptase using 2720 thermal cycler Singapore.

For miRNA 34 a gene expression, cDNA in a total reaction volume 20 μl and using small RNA RNU6B as an internal control. 10 ul 2X TaqMan® Universal Master mix, 1 ul TaqMan® miRNA Assay, 7 ul RNase-free water were pipetted into each PCR tube. 2 μl of cDNA was pipetted into each well and pipetted up and down two times to mix. Then the samples were incubated at 50 °C for 2 min and at 95 °C for 10 min. 40 cycles at 95 °C for 15 s and at 60 °C for 1 min using the 7500 Real-time PCR system (Applied Biosystems, Foster City, CA, USA).

**Fig. 1:**
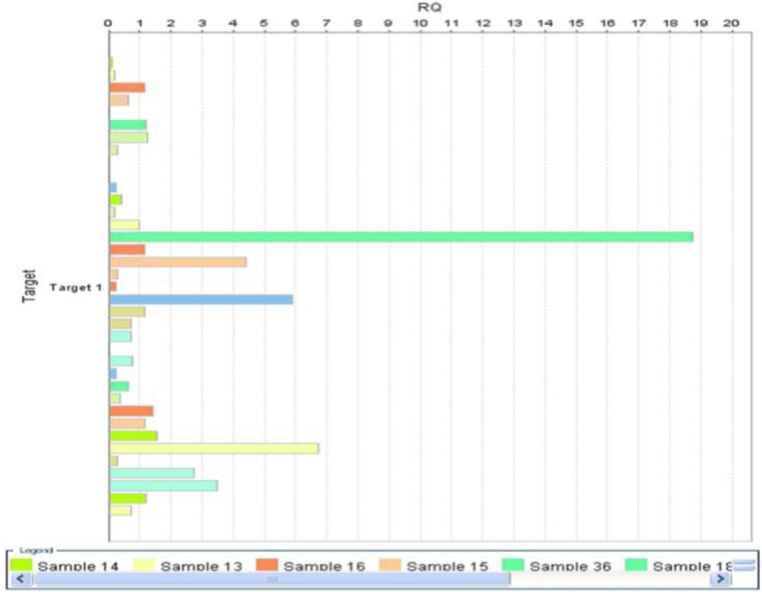
Gene epression plot (RQ vs Target of miRNA 34a gene expression.

**Fig. 2:**
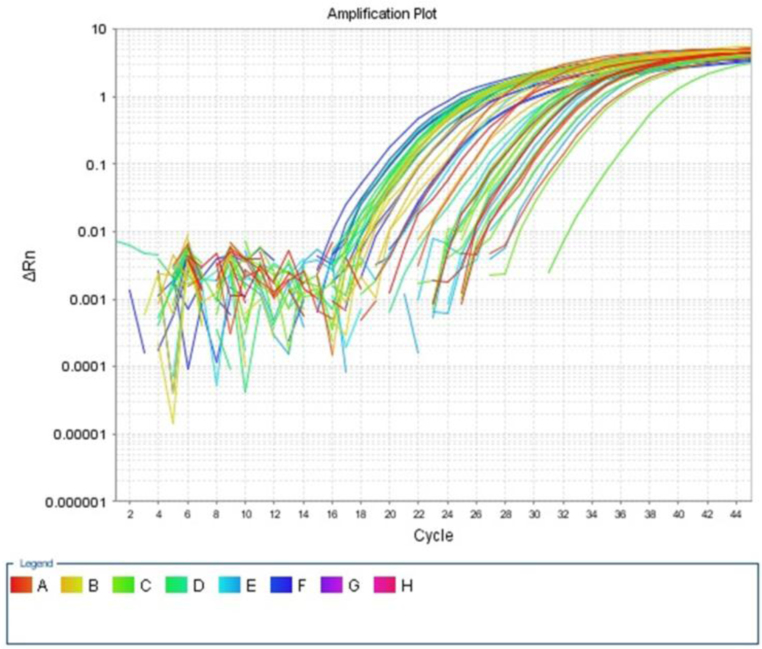
Amplification plot of miRNA34a gene expression.

The primers and TaqMan probes were designed by Applied Biosystems (Foster City, CA, USA) Life Technologies. Fig. 1: shows a Gene Expression Plot (RQ vs Target of miRNA 34a gene expression), Fig. 2: shows an Amplification plot of miRNA34a gene expression

For P53 gene expression

For reverse transcription step, samples were prepared in a final volume of 20 ul containing RT buffer, Multi scribe reverse transcriptase (PE Applied Biosystems), and 20 ng total RNA. Then the samples were incubated at 25 °C for 10 min and at 48 °C for 30 min. Heating to 95 °C for 5 min inactivated the reverse transcriptase on 2720 thermal cycler Singapore.

For cDNA amplification the sequence of primers of P53,

Forward primer AGTCTAGAGCCACCGTCCA Reverse primer TCTGACGCACACCTATTGCAAGC were used with SensiFAST™ SYBR® Lo-ROX Kit, nuclease-free water, cDNA in a total reaction volume 25 ul and using GAPDH as endogenous control using the 7500 Real-time PCR system (Applied Biosystems, Foster City, CA, USA).

**Fig. 3:**
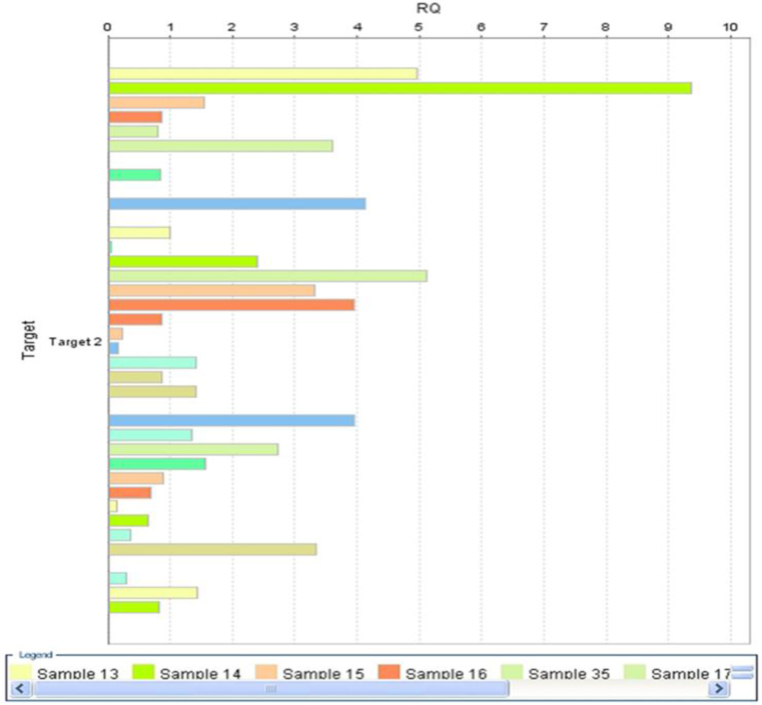
Gene expression plot (RQ vs Target of P53 gene expression.

Using the comparative CT method, you can use endogenous controls to normalize the expression levels of target genes by correcting differences in the amount of cDNA loaded into PCR reactions. Fig. 3: shows a Gene Expression Plot (RQ vs Target of P53 gene expression)

## 3 Statistical analysis

Qualitative data were described using number and percent and was compared using Chi-square test or Fisher Exact test, while normally quantitative data were expressed in mean ± SD and was compared using F test (ANOVA) abnormally distributed data was expressed in median (Min.–Max.) And was compared using Kruskal Wallis test.

Normally quantitative data were expressed in mean ± SD and was compared using F test (ANOVA) abnormally distributed data was expressed in median (Min.–Max.) and was compared using Kruskal Wallis test P-value ≤ 0.05 was considered statistically significant.

Receiver operator characteristic (ROC) with respective points of maximal accuracy for sensitivity and specificity were generated to determine biomarker performance. an area under the ROC curve (AUROC) Measures the accuracy of the test. An area of 1 represents a perfect test; an area of 0.5 represents a worthless test. 0.90–1 = excellent (A) 0.80–0.90 = good (B).

## 4 Results

This case-control study was conducted from January 2016 till February 2018 included 90 participants divided into 3 groups.

Group 1 included 30 colon cancer patients, group 2 included 30 patients with benign colonic polyps, and group 3 included 30 apparently normal individuals.

Regarding demographic features of the patients (age, gender, BMI) there was no statistically significant difference between the three groups indicating homogeneity between them (Table 1).

**Table 1:** Comparison between the different studied groups according to demographic data.

	**Cancer (n = 30)**	**Benign (n = 30)**	**Control (n = 30)**	**p**
**Sex**				
Male	12 (40%)	15 (50%)	15 (50%)	0.669
Female	18 (60%)	15 (50%)	15 (50%)	
**Age (years)**	52 ± 15.1	55.2 ± 16.5	52.1 ± 14.9	0.668
**BMI (kg/m**^**2**^**)**	26.7 (18.3–64.5)	25.5 (17.3–47.7)	26.2 (18.5–47.7)	0.474

Qualitative data were described using number and percent and was compared using Chi square test or Fisher Exact test, while normally quantitative data was expressed in mean ± SD and was compared using F test (ANOVA) abnormally distributed data was expressed in median (Min.–Max.) and was compared using Kruskal Wallis test.

Regarding baseline hemoglobin (Hb)%, patients with colon cancer (group 1) showed statistically significant lower level compared to the others two groups. However tumor markers (CA19.9 and CEA) serum levels were significantly elevated in colon cancer patients. ( Table 2)

**Table 2:** Comparison between the different studied groups according to different parameters.

	**Cancer (n = 30)**	**Benign (n = 30)**	**Control (n = 30)**	**p**	**Post hoc test (LSD or Dunn's)**		
					**Cancer vs. benign**	**Cancer vs. control**	**Benign vs. control**
**Hb% (gm/dl)**	9 ± 1.5	11.8 ± 0.7	11.9 ± 0.6	< 0.001*	< 0.001*	< 0.001*	0.739
**CEA mg/dl**	21 (9–43)	8 (5–12)	8 (5–12)	< 0.001*	< 0.001*	< 0.001*	0.860
**CA19–9 U/ml**	28.5 (10–51)	12 (9–15)	12 (9–15)	< 0.001*	< 0.001*	< 0.001*	0.709
**Gene expression of micro-RNA 34a**	1.2 (0.2–6.7)	16.1 (8.9–19.7)	16.7(11.8–20.1)	< 0.001*	< 0.001*	< 0.001*	0.530
**P53 gene expression**	1.4 (0.1–6.6)	10.3 (8.6–12.4)	10.8 (8.6–15.7)	< 0.001*	< 0.001*	< 0.001*	0.137

Normally quantitative data was expressed in mean ± SD and was compared using F test (ANOVA) abnormally distributed data was expressed in median (Min.–Max.) and was compared using Kruskal Wallis test.

* Statistically significant at p ≤ 0.05.

Regarding miRNA34a and p53 gene expression the colon cancer group showed statistically significant lower gene expression compared to the others two groups. (Table 2)

**Fig. 4:**
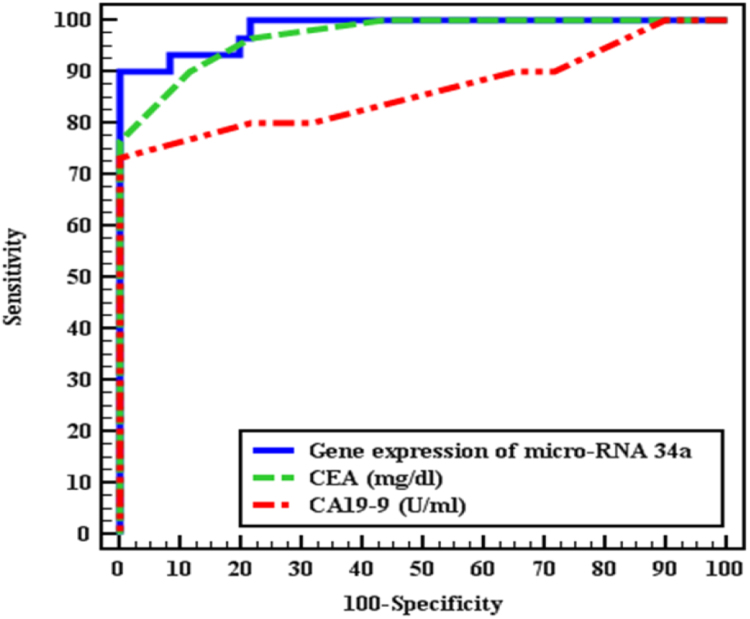
ROC curve of serum CEA, CA19-9 and miRNA34a gene expression.

To assess the diagnostic value of miRNA34a gene expression compared to CEA and CA19.9 serum levels, miRNA34a gene was both sensitive and specific for colon cancer (Table 3 and Fig. 4).

**Table 3:** Cutoff value, sensitivity, specificity and accuracy of gene expression of miRNA34a, CEA and CA19-9 levels to diagnosis CRC patients CRC patients from non-patients.

	**AUC**	**p**	**95% C.I**	**Cutoff**	**Sensitivity**	**Specificity**	**PPV**	**NPV**
**Gene expression of micro-RNA 34a**	0.983	<0.001^*^	0.962–1.00	≤5.4	90.0	91.67	84.4	94.8
**CEA mg/dl**	0.970^*^	<0.001^*^	0.940–1.0	>10	90.0	88.33	79.4	94.6
**CA19–9 U/ml**	0.864^*^	<0.001^*^	0.765–0.962	>15	73.33	100.0	100.0	88.2

Regarding the relation between micro RNA34a gene expression and different parameters in each group there was statistically significant relation between baseline serum tumor markers (CEA and CA19-9) and micro RNA34a gene expression in cancer patients as elevated tumor markers was associated with lower gene expression (Table 4).

**Table 4:** Correlation between micro RNA 34a gene expression and different parameters in each group (n = 30).

	**Micro RNA 34a gene expression**					
	**Cancer group**		**Benign group**		**Control group**	
	**r**_**s**_	**p**	**r**_**s**_	**p**	**r**_**s**_	**p**
**Age (years)**	0.070	0.715	−0.212	0.261	−0.121	0.524
**Weight (kg)**	−0.174	0.357	0.174	0.358	−0.124	0.514
**Height (cm)**	−0.257	0.171	0.387^*^	0.035^*^	−0.194	0.305
**BMI (kg/m**^**2**^**)**	−0.201	0.287	0.095	0.619	−0.046	0.809
**Neutrophil/Lymphocyte ratio**	−0.350	0.058	–	–	–	–
**Hb % (gm/dl)**	0.072	0.704	−0.099	0.601	−0.087	0.646
**Platelets (10^3^/mm^3^)**	−0.100	0.600	−0.339	0.067	0.103	0.589
**ALT (IU/L)**	0.150	0.429	−0.327	0.078	−0.065	0.734
**AST (IU/L)**	0.154	0.416	−0.235	0.211	0.346	0.061
**Urea (mg/dl)**	−0.120	0.529	−0.328	0.077	0.245	0.193
**Creatinine (mg/dl)**	−0.139	0.464	0.143	0.452	0.222	0.237
**CEA mg/dl**	−0.791^*^	<0.001^*^	0.050	0.794	−0.102	0.590
**CA19–9 U/ml**	−0.606^*^	<0.001^*^	0.035	0.855	−0.183	0.333
**P53 gene expression**	0.767^*^	<0.001^*^	0.672^*^	<0.001^*^	−0.023	0.904

Also there was statistically significant relation between micro RNA 34a gene expression and P53 gene expression in both colon cancer and benign colonic adenoma cases as the expression level of both genes were decreased. (Table 4).

Regarding the relation between P53 gene expression and other parameters in each groups, P53 gene expression had statistically significant relation with CEA and CA19-9 blood levels as low P53 gene expression was associated with elevated tumor markers in colon cancer group (Table 5).

**Table 5:** Correlation between P53 gene expression and different parameters in each group (n = 30).

	**P53 gene expression**					
	**Cancer group**		**Benign group**		**Control group**	
	**r**_**s**_	**p**	**r**_**s**_	**p**	**r**_**s**_	**p**
**Age (years)**	0.094	0.623	0.007	0.972	0.025	0.896
**Weight (kg)**	−0.231	0.219	0.308	0.098	0.352	0.056
**Height (cm)**	−0.146	0.440	0.340	0.066	−0.181	0.339
**BMI (kg/m**^**2**^**)**	−0.270	0.150	0.267	0.153	0.364^*^	0.048^*^
**Neutrophil/Lymphocyte ratio**	−0.280	0.134	–	–	–	–
**Hb % (gm/dl)**	0.168	0.373	−0.104	0.585	0.035	0.855
**Platelets (10^3^/mm^3^)**	−0.074	0.699	−0.239	0.203	0.095	0.619
**ALT (IU/L)**	0.038	0.842	−0.138	0.468	−0.067	0.726
**AST (IU/L)**	0.096	0.615	−0.157	0.407	−0.124	0.514
**Urea (mg/dl)**	−0.080	0.675	−0.319	0.086	−0.285	0.127
**Creatinine (mg/dl)**	−0.123	0.516	0.151	0.424	0.305	0.102
**CEA mg/dl**	−0.609^*^	<0.001^*^	0.014	0.943	−0.014	0.942
**CA19–9 U/ml**	−0.530^*^	0.003^*^	−0.031	0.870	−0.239	0.204

Regarding patients features in colon cancer group, 2 patients had positive family history of cancer (breast and colon cancer), carcinoma involving right ascending colon were more common than that involving left descending colon (16patients representing 36.7% versus 13 patients representing 33.3% of all patients) and only one case in transverse colon (Table 6).

**Table 6:** Distribution of the studied colon cancer cases according to different parameters in cancer group (n = 30).

		**No.**	**(%)**
**Family history**	No	28	(93.3%)
	Yes	2	(6.7%)
**Site**	Right	16	(53.3%)
	Left	13	(43.3%)
	Transverse	1	(3.33%)
**Presentation**	Abdominal pain	18	(60%)
	Constipation	3	(10%)
	Vomiting	0	(0%)
	Jaundice	0	(0%)
	Bleeding per rectum	6	(20%)
	Intestinal obstruction	3	(10%)
**Performance status:**	0	12	(40%)
	1	13	(43.3%)
	2	5	(16.7%)
**Pathology**	Signet ring	2	(6.7%)
	Mucinous	6	(20%)
**Grade**	I	3	(10%)
	II	19	(63.3)
	III	8	(26.7)
**Stage**	I	0	(0%)
	II	7	(23.3%)
	III	11	(36.7%)
	IV	12	(40%)
**Metastasis**	No	18	(60%)
	Yes	12	(40%)
**Site of metastasis (n = 12)**	Liver	4	(33.4%)
	Abdominal Lymph nodes	3	(25%)
	Liver and lung	5	(41.7%)
**L**/**N ratio**		2.9	(0.64–4.5)
**Initial response**	Regressive	8	(26.7%)
	Progressive	5	(16.7%)
	Stable disease	17	(56.6%)
**Time to progress (n = 14)**		5.5 ± 2.9	
**Overall survival (n = 14)**		7.5 (2–21)	
**Fate after 12 months**	Live	16	(53.3%)
	Dead	14	(46.7%)
**Cause of death (n = 14)**	Intestinal obstruction	5	(35.7%)
	Liver cell failure	5	(35.7%)
	Pulmonary embolism	1	(7.1%)
	Respiratory failure	2	(14.3%)
	Severe hypovolemic	1	(7.1%)

Regarding presenting symptoms abdominal pain was the most common presenting symptom in 60% of patients. Most of the patients have good performance status (performance 0 in 12 patients representing (40%) and 1 in 13 patients representing 43.3% of all patients) while 5 patients (16.7%) had performance status 2 at presentation (Table 6).

Regarding pathological subtypes only 2 9(6.7%) patients had signet ring differentiation and 6 (20%) patients had mucinous differentiation. Strikingly, no patient was presented with stage I disease, most patients was stage IV disease followed by stage III then stage II disease indicating tumor aggressiveness (Table 6).

Regarding metastatic sites liver was the most frequently involved site either alone in 4(33.4%) patients or with lung involvement in 5(43.7%) patients. the mean neutrophil and lymphocytic ratio in this group of patients was 2.9 (Table 6).

A time of initial evaluation, 5 patients experienced progressive disease, however at the end of follow up period 14 patients were dead 13 due to disease-related causes and 1 patient due to severe chemotherapy-induced diarrhea. The mean time to progression (TTP) of these patients was 5.5 ± 2.9 months and the median survival was7.5 months (Table 6).

Micro RNA 34a and P53 gene expression had statistically significant relation with tumor stage and presence of metastases as patients with stage IV disease had the lowest gene expression followed by stage III then stage II patients. Also presence of metastases was associated with lower gene expression ( Table 7 and 8).

**Table 7:** Relation between micro RNA 34a gene expression and different parameters in cancer group (n = 30).

	**N**	**Micro RNA 34a gene expression**			**Test of sig.**	**p**
		**Min.–Max.**	**Mean ± SD.**	**Median**		
**Metastasis**						
Present	**12**	0.25–1.17	0.75 ± 0.28	0.73	U = 27.50^*^	0.001^*^
Absent	**18**	0.20–6.74	2.87 ± 2.05	2.45		
**Fate**						
Live	**16**	1.14–6.74	3.22 ± 1.91	2.56	U = 2.00^*^	<0.001^*^
**Dead**	**14**	0.20–1.17	0.66 ± 0.30	0.71		
**Grade**						
I	**3**	2.56–6.45	4.47 ± 1.95	4.41	H = 5.168	0.075
II	**19**	0.20–6.74	1.88 ± 1.88	1.15		
III	**8**	0.30–3.65	1.43 ± 1.34	0.73		
**Stage**						
II	**7**	1.44–6.74	3.78 ± 2.16	2.56	H = 13.933^*^	0.001^*^
III	**11**	0.20–5.87	2.29 ± 1.85	1.55		
IV	**12**	0.25–1.17	0.75 ± 0.28	0.73

**Table 8:** Relation between P53 gene expression and different parameters in cancer group (n = 30).

	**N**	**P53 gene expression**			**Test of sig.**	**p**
		**Min.–Max.**	**Mean ± SD.**	**Median**		
**Metastasis**						
Present	**12**	0.15–1.55	0.75 ± 0.49	0.75	U = 25.00^*^	<0.001^*^
Absent	**18**	0.36–6.57	3.21 ± 2.06	3.33		
**Fate**						
Live	**16**	0.36–6.57	3.50 ± 2.0	3.64	U = 22.00^*^	<0.001^*^
**Dead**	**14**	0.15–1.55	0.77 ± 0.47	0.82		
**Grade**						
I	**3**	3.32–4.90	4.14 ± 0.79	4.20	H = 3.882	0.144
II	**19**	0.15–6.57	2.08 ± 1.95	1.43		
III	**8**	0.17–6.47	1.85 ± 2.27	0.84		
**Stage**						
II	**7**	0.86–6.57	3.76 ± 1.69	3.94	H = 12.956^*^	0.002^*^
III	**11**	0.36–6.47	2.85 ± 2.27	1.67		
IV	**12**	0.15–1.55	0.75 ± 0.49	0.75		

Patients with grade III tumors also had the lowest micro RNA 34a and P53 gene expression and patients with grade I tumors had the highest gene expression although this relation was statistically non significant (Table 7 and 8). Patients who experienced disease progression and died at the end of follow up period also experienced lower both micro RNA 34a and P53 gene expression than the patients who survived and this relation was statistically significant. (Table 7 and 8).

## 5 Discussion

MicroRNAs (miRs) are small noncoding RNAs that regulate gene expression by binding to the three un-translated regions of their target mRNAs. There are three homologs of miRNA34 (hsa-miRNA34a, hsa-miRNA34b and hsa-miRNA34c) in humans [12].

MiRNA34a acts as a tumor-suppressor gene by targeting many oncogenes related to proliferation, apoptosis and invasion [12].

Low expression of miRNA34a was detected in colon cancer tissues while over expression of miRNA34a inhibited colon cancer cell migration and invasion [15].

Both members of the miRNA34 family and p53 are related as the miRNA34 family direct p53 targets, and their up-regulation induces apoptosis and cell-cycle arrest [9].

In the current study 90 subjects were included in the study divided into three groups 30 subjects each. Group, I included patients with proved colon cancer, group II patients with proved colonic adenoma and group III included age and sex-matched apparently healthy individuals.

The three groups were homogenous regarding epidemiological features. However, in laboratory parameters patients in cancer group had significantly lower Hb% than in the other groups this can be explained by chronic blood loss from the malignant tumor.

Serum Tumor markers CEA and CA19-9 were significantly elevated in patients in colon cancer group compared to other groups indicating their diagnostic value.

This is different from that was reported by Polat et al. who reported Serum CA 19-9 was not significantly different in the control and patient groups while serum CEA was significantly higher in the patient group than in the control group [16].

Regarding miRNA34a gene expression within the studied groups, the colon cancer group showed statistically significant lower gene expression compared to patients with adenomatous polyps and healthy controls.

This accordance with the study of Ma et al. who found that Polyps tissues had significantly higher miRNA34a gene expression than para-neoplastic and colorectal cancer tissue samples [17].

Also, Wu et al. and Tazawa et al. reported that miRNA34a gene expression was down-regulated in colon cancer tissue samples [15], [18].

While, Aherne et al. studied the expression of 667 miRNAs in plasma samples of a discovery cohort of patients with benign and malignant disease of the colon compared to age and sex-matched disease-free controls a group and found that MiRNA34a expression was significantly increased in the adenoma and early-stage cancer groups compared to healthy controls, and moderately in the advanced cancer group [19].

P53 gene is a proved tumor suppressor gene that is connected to many types of cancers in the current study it was down-regulated in colon cancer patients and this is consistent with malignant transformation.

In order to assess the validity of MiRNA34a expression in the diagnosis of colon cancer, Receiver operator characteristic (ROC) curve was performed and respective points of maximal accuracy for sensitivity and specificity were generated and it was found that MiRNA34a expression was sensitive and specific tests in the diagnosis of colon cancer.

These data are similar to what was found by Aherne et al. who reported that MiRNA34a expression in plasma samples can distinguish normal group from cancer group as miRNA34a expression was significantly decreased in early-stage cancer samples compared to the non-malignant patient samples [18].

So theoretically down-regulation of miRNA34a gene expression can be a mean for diagnosing malignant transformation in patients with the chronic inflammatory condition in the colon like ulcerative colitis and Chron's disease instead of frequent colonoscopy assessment and other invasive tools.

Regarding the relation between miRNA34a gene expression and p53 gene, in the current study, they were both down-regulated in the colon cancer patients.

Matched with these results, Wu et al. also studied this relation in colon cancer tissue specimens and found that DNA-binding activity of p53 was significantly correlated with miRNA34a expression in colon cancer tissues [15].

Likewise, miRNA34a deficiency present in colorectal cancer tissue leads to mutation and deficiency of P53 which accelerate cancer progression [20], [21].

Mortality from cancer mostly attributes to metastases of this cancer, not to the primary tumors itself. This may be explained by the resistance of disseminated tumor cells to therapeutic agents [22].

In the current study, the authors investigated the relation between disease parameters in colon cancer patients which had not been investigated before we found that both miRNA34a gene expression and p53 gene expression were down regulated in patients with stage IV disease, grade III pathology and in patients with shorter time to progression. Indicating that: both miRNA34a and p53 genes are directly connected to tumor aggressiveness and patients' outcomes.

The study of Wu et al. explained the relation between miRNA34a gene expression level and metastasis of colorectal cancer by forming activator protein-1 (AP-1) heterodimers through targeting FRA1, a FOS transcription factor [15].

While, Rokavec et al. reported that miRNA34a can cause inhibition of metastasis due to tumor invasion or migration by negative influence on the IL-6/STAT3 signaling pathway [23].

It could conclude that the more the reduction of miRNA34a and P53 gene expression the more the aggressiveness and progress of colon cancer. The level of miRNA34a can be used to differentiate between colon cancers and begin adenoma. MiRNA34a may have a beneficial role in colon cancer.

## References

[1] Ferlay J., Soerjomataram I., Dikshit R., Eser S., Mathers C., Rebelo M. (2015). Cancer incidence and mortality worldwide: sources, methods and major patterns in GLOBOCAN 2012. Int. J. Cancer.

[2] Burch J.A., Soares-Weiser K., St John D.J., Duffy S., Smith S., Kleijnen J. (2007). Diagnostic accuracy of faecal occult blood tests used in screening for colorectal cancer: a systematic review. J. Med. Screen..

[3] Chen X., BaY Ma.L., Cai X., Yin Y., Wang K. (2008). Characterization of microRNAs in serum: a novel class of biomarkers for diagnosis of cancer and other diseases. Cell Res..

[4] He L., Hannon G.J. (2004). microRNA: small RNAs with a big role in gene regulation. Nat. Rev. Genet..

[5] Esquela-Kerscher A., Slack F.J. (2006). Oncomirs - microRNAs with a role in cancer. Nat. Rev. Cancer.

[6] Ebner O.A., Selbach M. (2014). Quantitative proteomic analysis of gene regulation by miR-34a and miR-34c. PLoS One.

[7] Tarasov V., Jung P., Verdoodt B., Lodygin D., Epanchintsev A., Menssen A., Meister G., Hermeking H. (2007). Differential regulation of microRNAs by p53 revealed by massively parallel sequencing: miR-34a is a p53 target that induces apoptosis and G1-arrest. Cell Cycle.

[8] Gao J., Li N., Dong Y., Li S., Xu L., Li X., Li Y., Li Z., Ng S.S., Sung J.J. (2015). miR-34a-5p suppresses colorectal cancer metastasis and predicts recurrence in patients with stage II/III colorectal cancer. Oncogene.

[9] Hermeking H. (2010). The miR-34 family in cancer and apoptosis. Cell Death Differ..

[10] Li Y., Guessous F., Zhang Y., Dipierro C., Kefas B., Johnson E., Marcinkiewicz L., Jiang J., Yang Y., Schmittgen T.D. (2009). MicroRNA-34a inhibits glioblastoma growth by targeting multiple oncogenes. Cancer Res..

[11] Taketo M.M. (2011). Reflections on the spread of metastasis to cancer prevention. Cancer Prev. Res..

[12] Li X., Ren Z., Tang J. (2014). MicroRNA-34a: a potential therapeutic target in human cancer. Cell Death Dis..

[13] Eisenhauer E., Therasse P., Bogaerts J., Schwartzd L., Sargente D., Ford R. (2009). New response evaluation criteria in solid tumours: revised RECIST guideline (version 1.1). Eur. J. Cancer.

[14] Oken M.M., Creech R.H., Tormey D.C., Horton J., Davis T.E., McFadden E.T., Carbone P.P. (1982). Toxicity and response criteria of the eastern cooperative oncology group. Am. J. Clin. Oncol..

[15] Wu J., Wu G., Lv L., Ren Y.F., Zhang X.J., Xue Y.F. (2012). MicroRNA-34a inhibits migration and invasion of colon cancer cells via targeting to Fra-1. Carcinogenesis.

[16] Polat E., Duman U., Duman M., Atici A., Reyhan E., Dalgic T., Bostanci E., Yol S. (2014). Diagnostic value of preoperative serum carcinoembryonic antigen and carbohydrate antigen 19-9 in colorectal cancer. Curr. Oncol..

[17] Ma Z., Kong X., Cui G., Ren C., Zhang Y., Fan S., Li Y. (2014). Expression and clinical significance of miRNA-34a in colorectal cancer. Asian Pac. J. Cancer Prev..

[18] Tazawa H., Tsuchiya N., Izumiya M., Nakagama H. (2007). Tumor-suppressive miR-34a induces senescence-like growth arrest through modulation of the E2F pathway in human colon cancer cells. Proc. Natl. Acad. Sci. USA.

[19] Aherne S., Madden S., Hughes D., Pardini B., Naccarati A., Levy M., Vodicka P., Neary P., Dowling P., Clynes M. (2015). Circulating miRNAs miR-34a and miR-150 associated with colorectal cancer progression. BMC Cancer.

[20] Leedham S.J., Graham T.A., Oukrif D., McDonald S.A., Rodriguez-Justo M., Harrison R.F., Shepherd N.A., Novelli M.R., Jankowski J.A., Wright N.A. (2009). Clonality, founder mutations, and field cancerization in human ulcerative colitis-associated neoplasia. Gastroenterology.

[21] Okada N., Lin C.-P., Ribeiro M.C., Biton A., Lai G., He X., Bu P., Vogel H., Jablons D.M., Keller A.C. (2014). A positive feedback between p53 and miR-34 miRNAs mediates tumor suppression. Genes Dev..

[22] Valastyan S., Weinberg R.A. (2011). Tumor metastasis: molecular insights and evolving paradigms. Cell.

[23] Matjaz Rokavec, Gulfem Meryem Oner, Huihui Li.Heiko, Hermeking (2014). IL-6R/STAT3/miR-34a feedback loop promotes EMT-mediated colorectal cancer invasion and metastasis. J. Clin. Investig..

