# Supplementary material for: The clinical impact of miRNA34a and P53 gene expression in colon cancer
Source: Biochem Biophys Rep. 2018 Oct 24;16:88–95. doi: 10.1016/j.bbrep.2018.10.002 (PMC6202666; doi:10.1016/j.bbrep.2018.10.002)
Supplement: Supplementary file 1 — Supplementary material [file mmc1.doc]

Dear Editor,

I confirm that all authors have no conflict of interest

Paper title

**The clinical impact of miRNA34a and P53 gene expression in colon cancer**

Eman AE Badr*, Mohamed Farag Ali Assar **, Suzy F Gohar***, Mohamed Hamdy Badr ****, Rawda Magdy Ahmed Hathout***** and Salah Mohamed El-kousy ******

Department of Medical Biochemistry, Faculty of Medicine - Menoufia University*

Department of Chemistry, Biochemistry Division, Faculty of science - Menoufia University, Shebin El koom, Egypt**

Department of Clinical Oncology, Faculty of Medicine - Menoufia University***

Department of Internal Medicine, Faculty of Medicine - Menoufia University****

Chemist at Faculty of Science – Menoufia University*****

Department of Organic chemistry, Faculty of science - Menoufia University******

Yours faithfully

Eman Badr

Professor, Faculty of Medicine

Menoufia University
